# Sintilimab for the treatment of lung adenocarcinoma-induced immune-related hypophysitis: a case report

**DOI:** 10.3389/fimmu.2025.1534179

**Published:** 2025-01-23

**Authors:** Ming-xing Wang, Ai-xin Liu, Qing-ming Sun, Wan-hui Dong

**Affiliations:** ^1^ Department of Medical Oncology, Lu’an Hospital of Traditional Chinese Medicine Affiliated to Anhui University of Chinese Medicine, Lu’an, China; ^2^ Department of Orthopedics Department, Lu’an Hospital of Traditional Chinese Medicine Affiliated to Anhui University of Chinese Medicine, Lu’an, China; ^3^ Department of Medical Oncology, Lu’an Hospital of Traditional Chinese Medicine, Lu’an, China

**Keywords:** sintilimab, hypophysitis, diabetes insipidus, adverse reactions, immune checkpoint inhibitors

## Abstract

Immune checkpoint inhibitors (ICIs) are extensively utilized in the treatment of oncological patients, and the immune-related adverse reactions they induce merit close monitoring. This report describes a case of a lung cancer patient who, after receiving chemotherapy in combination with the programmed death-1 (PD-1) inhibitor sintilimab, presented with systemic fatigue, alterations in mental and behavioral patterns, somnolence, and symptoms of diabetes insipidus, leading to a diagnosis of grade 4 immune-related hypophysitis. The patient experienced symptomatic relief following pulse therapy with dexamethasone sodium phosphate injections (30mg every 12 hours), and was subsequently treated with prednisone acetate tablets (30 mg/day), which were gradually reduced to a physiological replacement dose. The treatment with sintilimab was discontinued, and the patient’s symptoms gradually improved, with normalization of urine output.

## 1 Introduction

In recent years, immune checkpoint inhibitors (ICIs) have made significant therapeutic advancements in the treatment of non-small cell lung cancer (NSCLC), effectively prolonging patient survival and improving their quality of life. However, while ICIs activate the immune system to combat tumors, they can also potentially cause damage to multiple organ systems in patients. Among these, endocrine system side effects are particularly common, and the endocrine glands’ susceptibility may be related to the high vascular distribution characteristics of glands such as the pituitary, thyroid, and adrenal glands (1). Immune-related hypophysitis (irH), as a relatively difficult-to-diagnose endocrine system adverse reaction, can present with a variety of clinical manifestations, including headaches, visual impairments, and symptoms of pituitary dysfunction (2). Literature reports indicate that the incidence of immune-related hypophysitis varies widely, ranging from 1.8% to 18.3%, and may be related to factors such as patient age, gender, drug dosage, and type (3).

Sintilimab, as a novel PD-1 inhibitor, has shown good efficacy and safety in the treatment of lung adenocarcinoma. However, with the increase in clinical application, cases of hypophysitis induced by sintilimab have also been increasingly reported (4), presenting new challenges for clinicians in terms of diagnosis and treatment. The purpose of this article is to detail, through a case report, the clinical manifestations, diagnostic process, treatment strategies, and prognosis of a lung adenocarcinoma patient who developed hypophysitis following treatment with sintilimab, with the aim of enhancing clinicians’ awareness and management capabilities regarding such adverse reactions.

## 2 Case report

### 2.1 Patient’s chief complaint, past medical history, personal history, physical examination, and auxiliary examinations

A 61-year-old male patient was admitted to the hospital with the complaint of “having been diagnosed with lung cancer for over six months, and experiencing facial palsy for three days.” The patient has a history of smoking for over 30 years, consuming 2 packs daily. He denies a history of hypertension, diabetes, coronary heart disease, surgery, trauma, and exposure to epidemic water or areas. He is married with children, all in good health, except for his second son who passed away from lung cancer. Upon admission, physical examination revealed: T 36.5℃, P 83 beats per minute, R 19 breaths per minute, BP 110/87 mmHg, height 170 cm, weight 70 kg, body surface area 1.82 m², Karnofsky Performance Status (KPS) 70, Numerical Rating Scale (NRS) 5. The patient is conscious, appears lethargic, cooperates with the examination, and responds appropriately. There is no jaundice of the skin or mucous membranes, no petechiae or ecchymosis. No superficial lymph node enlargement is palpable. The head is normocephalic, lips without cyanosis, no abnormalities in the eyes, ears, or nose, no jugular venous distension, the neck is supple, trachea is midline, and both thyroid lobes are not enlarged. Breath sounds in both lungs are clear, with no crackles or wheezes. The heart rhythm is regular, with no murmurs. The abdomen is soft, with no abnormalities palpable, no lower limb edema, and no pathological reflexes in the nervous system. Laboratory tests revealed the following levels: D-dimer at 8.81 μg·ml^–1^ (normal range: <0.5 μg·ml^–1^), fibrin degradation product at 16.11 μg·ml^–1^ (normal range: <5 μg·ml^–1^). Tumor marker tests for squamous cell carcinoma indicated elevated levels: neuron-specific enolase at 55.83 ng·ml^–1^ (normal range: <16.3 ng·ml^–1^), cytokeratin 19 fragment at 187.81 ng·ml^–1^ (normal range: <3.3 ng·ml^–1^), carcinoembryonic antigen at 8184.54 ng·ml^–1^ (normal range: <5 ng·ml^–1^), and carbohydrate antigen at 6421.68 U·ml^–1^ (normal range: <35 U·ml^–1^). Liver function tests showed: alanine aminotransferase at 48.58 U·L^–1^ (normal range: 5-40 U·L^–1^), aspartate aminotransferase at 43.28 U·L^–1^ (normal range: 8-40 U·L^–1^), albumin-to-globulin ratio at 1.41 (normal range: 1.5-2.5), alkaline phosphatase at 155.44 U·L^–1^ (normal range: 40-150 U·L^–1^), and gamma-glutamyl transferase at 54.62 U·L^–1^ (normal range: 11-50 U·L^–1^). Magnetic resonance imaging (MRI) revealed: multiple nodules of varying sizes in both cerebellar hemispheres, both cerebral hemispheres, brainstem, basal ganglia, thalamus, and outside the brain, some with ring-like signals and unclear boundaries, with the largest one located in the left posterior parietal area outside the brain, measuring approximately 24mm x 21mm x 15mm, showing significant uneven enhancement, and the adjacent meninges are markedly thickened and enhanced, some arterial-enhancing small nodules are seen in the skull, the midline structures are centered, the ventricular system is not significantly dilated, and the brain sulci and fissures are widened and deepened. The diagnosis is multiple metastatic tumors in both the brain, outside the brain, and the skull, with focal meningeal involvement.

### 2.2 Patient’s current medical history

**Figure 1:**
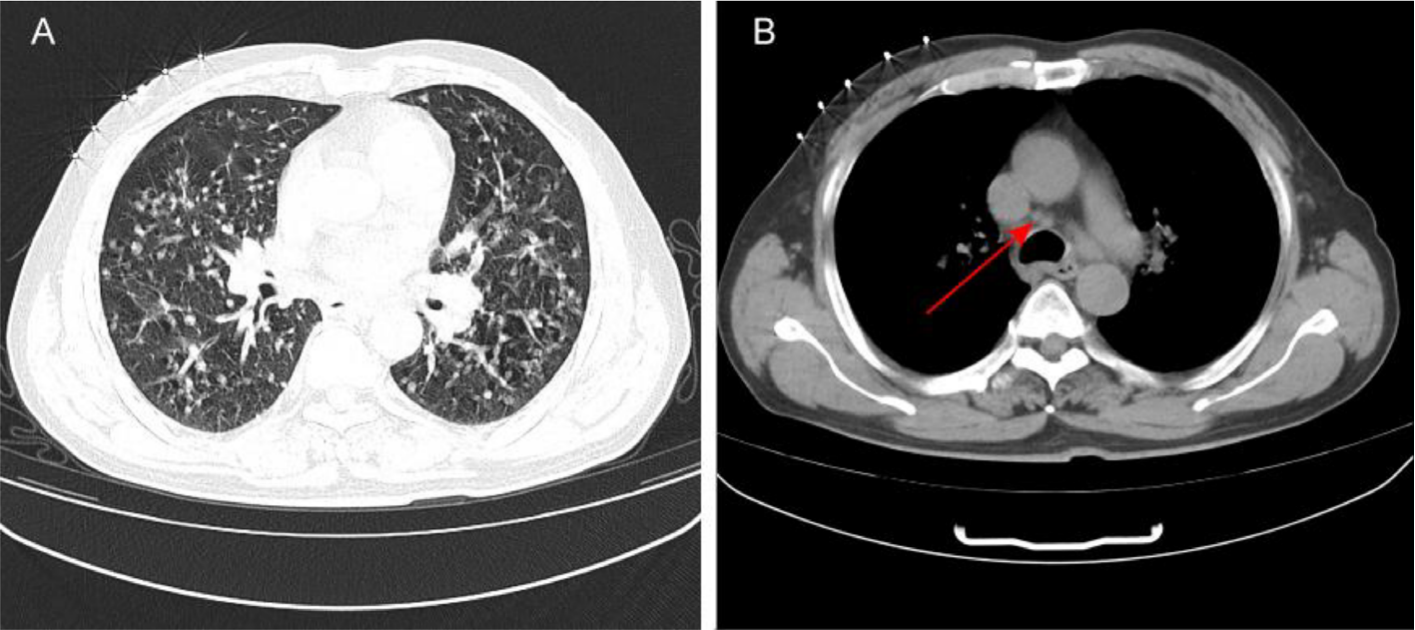
**(A)** The patient’s initial CT scan shows diffuse nodules in the lungs. **(B)**: The patient’s initial CT scan indicates enlarged mediastinal lymph nodes.

**Figure 2:**
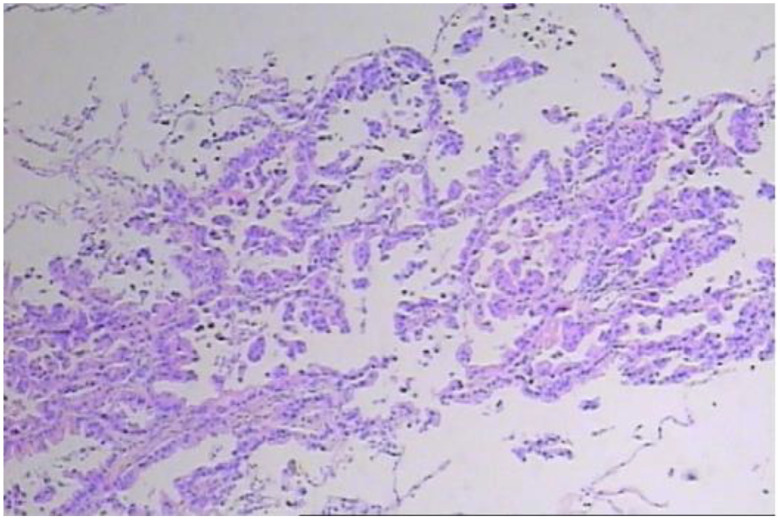
Lung biopsy pathology suggests adenocarcinoma with neuroendocrine differentiation.

In early March 2024, a 61-year-old male patient was admitted for a persistent cough with white sputum, which had been ongoing for three months without any identifiable cause. He denied experiencing hemoptysis, chest pain, or tightness. Despite only slight symptomatic relief from anti-inflammatory drugs at a community hospital, a CT scan on March 22, 2024, revealed diffuse nodules in both lungs and enlarged lymph nodes, suggesting a malignant tumor (**Figure 1**). A lung biopsy on March 28, 2024, confirmed non-small cell lung cancer, specifically adenocarcinoma with neuroendocrine differentiation, based on Immunohistochemistry (IHC) (**Figure 2**). The results were as follows: IHC: p40 (–), CK5/6 (–), TTF-1 (+), NapsinA (+), Syn (–), CD56 (+), CK7 (+), Ki-67 (+, 65%). Genetic testing on the biopsy specimen (ID: M01240405469) on April 22, 2024, showed no mutations in PD-L1, EGFR, ALK, ROS-1, RET, C-MET, or HER-2. Given the neuroendocrine features, the patient was initiated on 200 mg Sulfatinib daily. A follow-up CT on May 14, 2024, showed lung lesion improvement, but an ECT revealed multiple bone destructions, leading to a second course of Sulfatinib with phosphates to prevent further bone damage. By July 2024, after two Sulfatinib courses, tumor markers and lung lesions had progressed. The patient then received pemetrexed combined with cisplatin and bevacizumab on July 8, followed by two courses of docetaxel with carboplatin due to lack of significant improvement in tumor markers and symptoms. On October 2, 2024, the patient began anlotinib targeted therapy. However, on October 16, 2024, he was readmitted with facial palsy and severe headache for three days.

### 2.3 The patient’s treatment process

**Figure 3:**
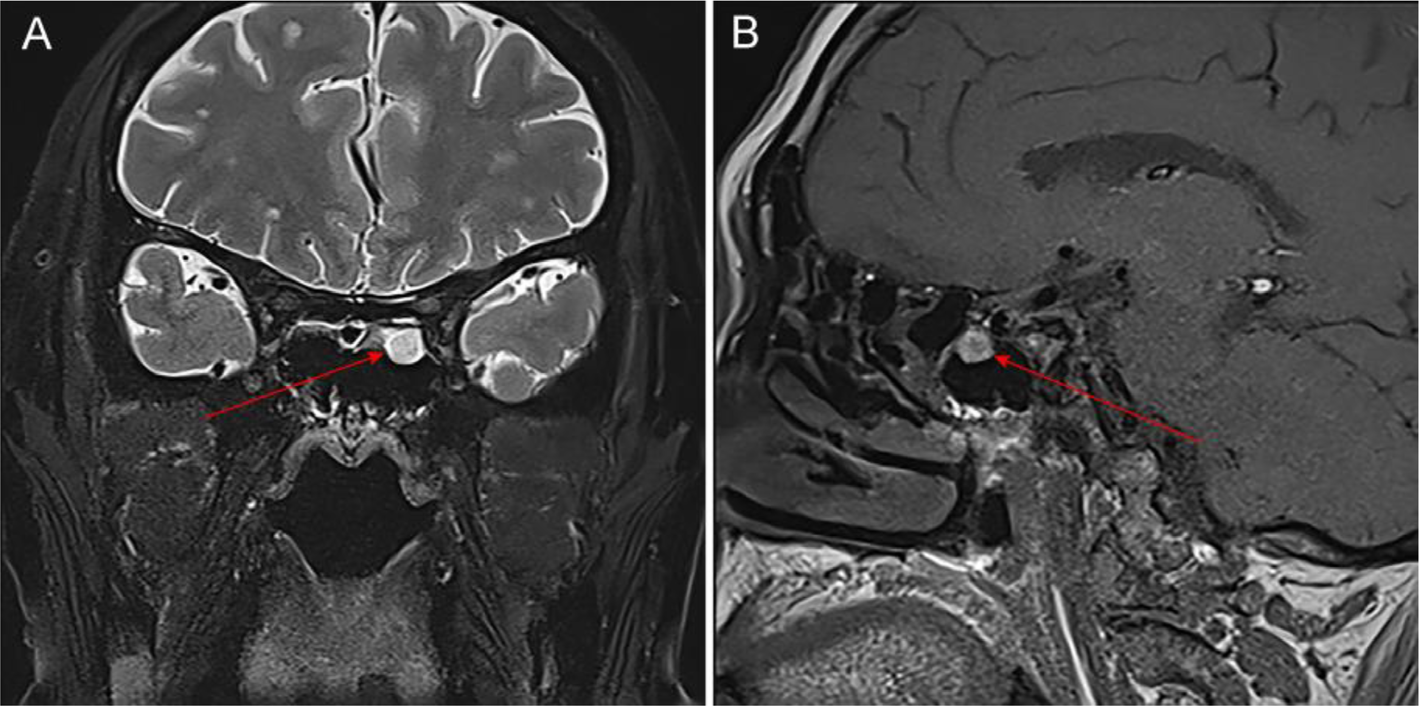
Imaging changes of the pituitary gland in the patient after using Sintilimab. **(A)** shows the coronal view of the pituitary T2 signal, and **(B)** shows the sagittal view of the pituitary T1 signal. Description of the pituitary MRI with plain and contrast: The sella turcica has a regular shape with no depression of the sellar floor, and no significant abnormal signal in the bone of the sella, there is a slight exudate-like change around the pituitary, about 7mm in height, and a nodule-like isointense slightly long T1 and slightly long T2 signal can be seen on the left side of the pituitary, which shows mild enhancement (lower than the adjacent normal pituitary tissue), with a long diameter of about 4mm, the pituitary stalk is slightly deviated to the left, with no significant abnormal signal. The bilateral cavernous sinus structure is clear, and the signal is not significantly abnormal, there is no obvious sign of compression and upward displacement of the optic chiasm, a circular short T1 and slightly long T2 signal can be seen in the left sphenoid sinus, which shows high signal on enhancement, with a clear edge; multiple isointense slightly long T1 and slightly long T2 signals can be seen in the brain tissue, with ring-like and nodular enhancement.

**Figure 4:**
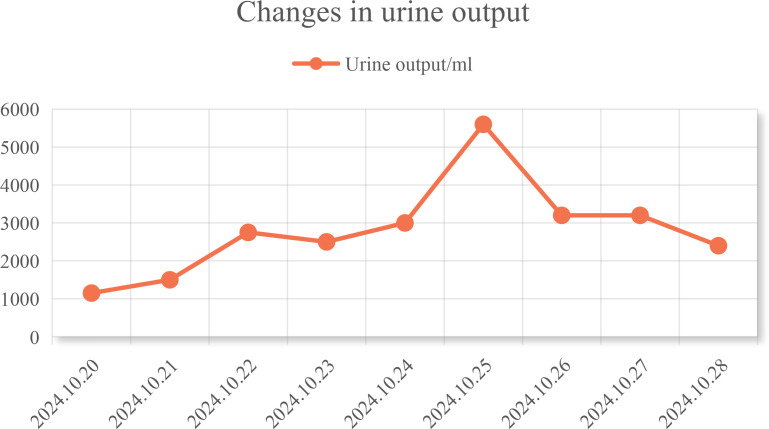
Changes in the patient’s urine volume.

**Figure 5:**
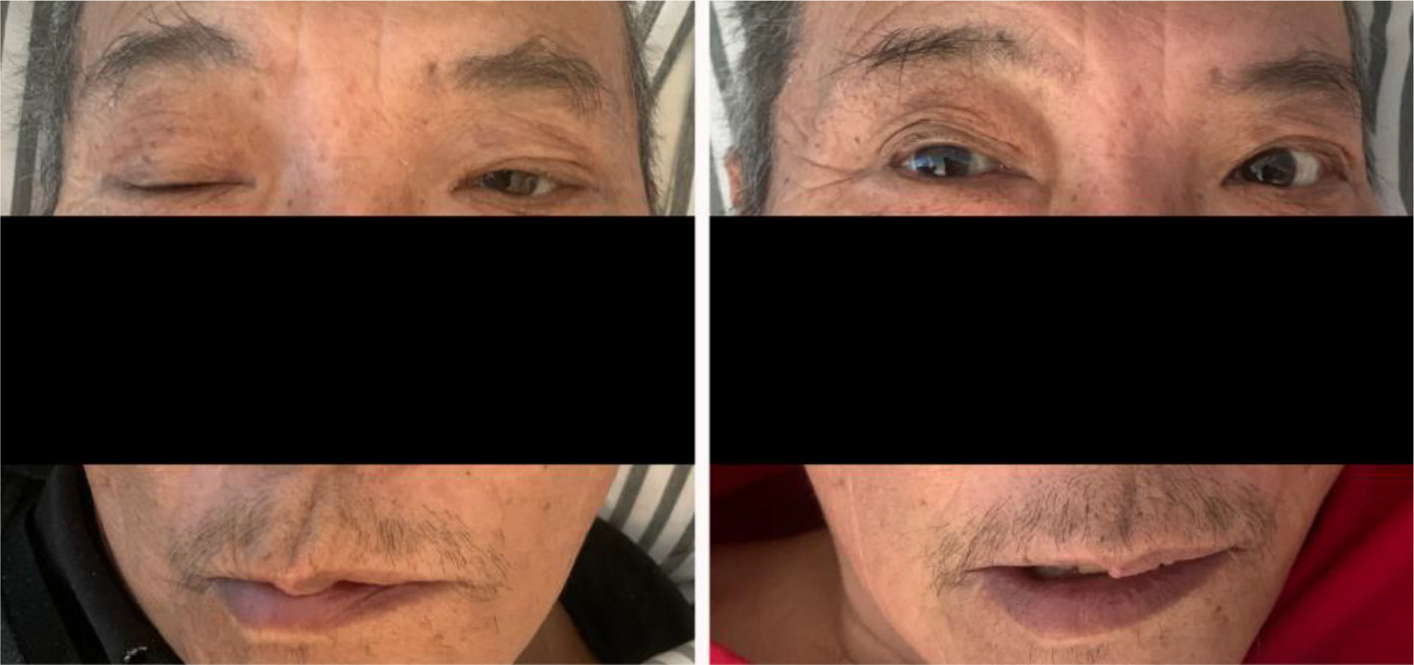
Changes in the patient’s facial appearance before and after steroid treatment (left image is before treatment, right image is after treatment).

**Figure 6:**
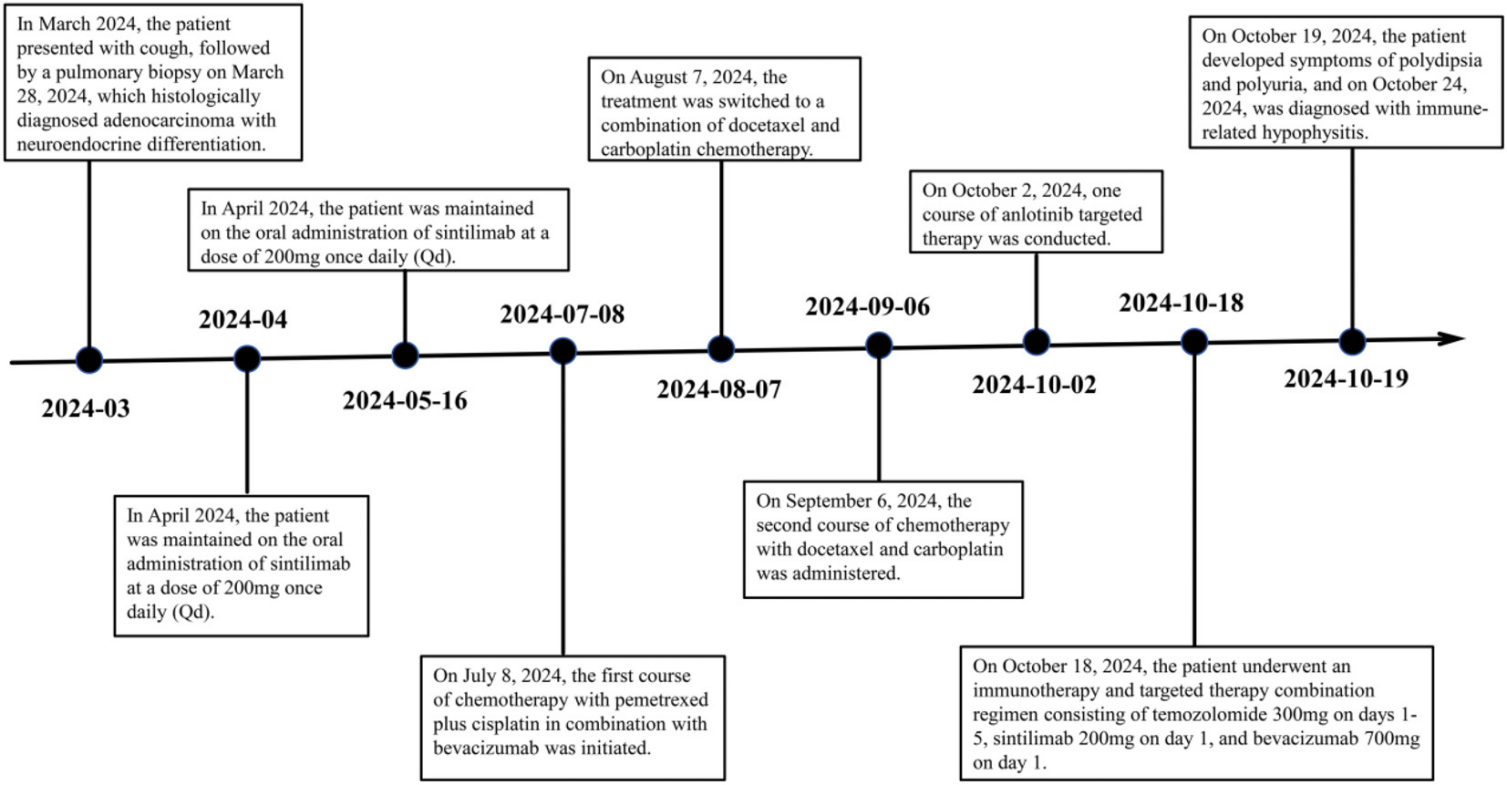
Timeline of the patient’s treatment.

In conjunction with the patient’s symptoms and auxiliary examination data, multiple intracranial metastases were confirmed. Considering the patient’s physical condition, on October 18, 2024, a combined immunotherapy and targeted treatment regimen of “Temozolomide 300mg D1-5 + Sintilimab 200mg D1 + Bevacizumab 700mg D1” was administered. On October 19, 2024, the patient reported severe thirst after the treatment on October 18, drinking 3000ml of water in half a day. The patient was instructed to record the 24-hour urine volume, and on October 22, the patient’s urine volume was 2750ml. A review of the inpatient biochemistry revealed: Alanine aminotransferase (ALT) was 47.97 U·L^–1^ (normal range: 5-40 U·L^–1^), Aspartate aminotransferase was 166.82 U·L^–1^ (normal range: 8-40 U·L^–1^), Urea was 1.51 mmol·L^–1^ (normal range: 3.1-8.2 mmol·L^–1^), Sodium was 146.34 mmol·L^–1^ (normal range: 136-145 mmol·L^–1^), Chloride was 110.45 mmol·L^–1^ (normal range: 96-108 mmol·L^–1^). Pituitary prolactin was 24.67 ng·ml^–1^ (normal range: 3.5-19.5 ng·ml^–1^), blood cortisol was 181.03 nmol·L^–1^ (normal range: 138-635 nmol·L^–1^), adrenocorticotropic hormone (ACTH) was 111.66 ng·L^–1^ (normal range: 7.2-63.3 ng·L^–1^), and urine specific gravity was 1.005 (normal range: 1.01-1.03). A magnetic resonance scan with plain and enhanced imaging of the pituitary gland indicated abnormal signal on the left side of the pituitary, diagnosing pituitary metastasis (see **Figure 3**). At this time, it was the 5th day after the use of Sintilimab, and based on the auxiliary examinations and radiological data, a diagnosis of secondary immune-related hypophysitis was made. On October 24, 2024, the patient was treated with Dexamethasone sodium phosphate injection 30mg every 12 hours. After 2 days of use, the urine volume decreased from 5600ml to 3200ml (see **Figure 4**), and the patient’s thirst symptoms and facial palsy showed slight improvement. On October 27, 2024, Prednisone acetate tablets 140mg were taken once daily, and on October 29, ACTH was 3.29 ng·L^–1^ (normal range: 7.2-63.3 ng·L^–1^). The patient’s vital signs were stable, and discharge was arranged with instructions to halve the medication dose every week. A follow-up phone call after half a month, the family reported that the urine volume had returned to normal (see **Figure 5** for changes in the patient’s face before and after treatment, and **Figure 6** for the treatment course).

## 3 Discussion

Recent literature reports indicate that sintilimab has a good antitumor effect (5), and it has shown similar antitumor effects and better safety profiles compared to nivolumab and pembrolizumab in Hodgkin’s lymphoma, NKT-cell lymphoma, and advanced non-small cell lung cancer (6). Sintilimab can block the interaction between PD-1 and its ligands, helping T cells to restore their antitumor function. However, due to its lack of tumor tissue specificity (7), it can damage T cells’ immune tolerance to their antigens, leading to a series of immune-related adverse events (iAEs) (8). In this case, the patient developed hypophysitis and diabetes insipidus after receiving chemotherapy and sintilimab immunotherapy for lung adenocarcinoma with brain metastasis. These conditions were promptly corrected with hormone replacement therapy, stabilizing pituitary function.

### 3.1 Definition, clinical manifestations, and differential diagnosis of immune-related hypophysitis

Immune-related hypophysitis is a rare form of hypophysitis and is the most common iAE associated with ICIs, typically occurring around the 8th to 10th week after starting ICIs (9). Clinical manifestations are mainly characterized by pituitary endocrine abnormalities or inflammatory compression, with typical symptoms including headache, nausea, fatigue, vision changes, and hypotension. Other less common clinical manifestations include mental confusion, memory loss, anorexia, hyponatremia, decreased sexual function, amenorrhea, and diabetes insipidus (10, 11). Due to the complex clinical presentation of hypophysitis patients and the similarity to symptoms caused by tumor progression, it is important to differentiate it from hypophysitis caused by other reasons such as infection or tumor (12). Additionally, it should be distinguished from other space-occupying lesions like pituitary adenomas, craniopharyngiomas, and Rathke’s cleft cysts. Complications arising from pituitary dysfunction may include secondary hypothyroidism, hypogonadotropic hypogonadism, and secondary adrenal insufficiency, with incidence rates of 7.6%, 7.5%, and 6.1%, respectively (13). Furthermore, there are various changes in adrenocorticotropic hormone (ACTH), TSH, growth hormone, and prolactin (14). These can be differentiated in conjunction with the patient’s gender, age, pituitary imaging, and laboratory tests.

### 3.2 Mechanism of action and common adverse reactions of sintilimab

Sintilimab, an immune checkpoint inhibitor and a recombinant fully human IgG monoclonal antibody against PD-1, binds to PD-1, blocks its interaction with PD-L1 and PD-L2, restores endogenous antitumor T-cell responses, and exerts antitumor effects (15). Approved by China National Medical Products Administration in 2018 for relapsed or refractory classical Hodgkin’s lymphoma after at least two lines of systemic chemotherapy. In 2021, sintilimab was granted additional indications for use in combination with other drugs for the first-line treatment of unresectable advanced or recurrent squamous cell carcinoma. Studies have reported that immune-related hypophysitis is due to excessive immune activation and represents an autoimmune reaction. CTLA-4 and PD-1 are immune checkpoint molecules found on T lymphocytes that inhibit T-cell activity (16). Anti-PD-1/PD-L1 therapy can enhance reactive T-cell activity in the pituitary, thereby mediating immune-related hypophysitis. The instructions for Sintilimab list common adverse reactions such as immune-related pneumonia, colitis, hepatitis, nephritis, and endocrine disorders, which require clinical vigilance (17).

### 3.3 Drug adverse reaction association assessment

After admission, the patient was diagnosed with lung adenocarcinoma and brain metastasis. Given the patient’s condition, a chemotherapy regimen of sintilimab + bevacizumab + temozolomide was selected. The patient had severe thirst on the second day of treatment, drinking 3000ml of water in half a day, and on the eighth day after chemotherapy, the urine volume reached 5600ml, leading to a diagnosis of diabetes insipidus. Concurrent lab tests showed abnormal pituitary prolactin and blood cortisol levels, and MRI showed abnormal signals in the pituitary area with exudate-like changes. Based on symptoms and test data, the patient was diagnosed with immune-related hypophysitis. The association between sintilimab and the patient’s immune-related hypophysitis was evaluated (**Table 1**) (1): The patient had no history of hypophysitis or pre-existing immune system-related diseases, and no other immunomodulatory drugs were used, excluding the immune system itself and past medical history as causes. The patient developed thirst and excessive drinking on the second day of chemotherapy with the sintilimab + bevacizumab + temozolomide regimen, showing a strong temporal association (2). The patient had facial palsy on admission, and MRI excluded cerebral infarction while diagnosing multiple intracranial metastatic tumors. Sintilimab can activate the immune system to attack tumor cells but may also cause immune cells to attack normal cells, triggering an autoimmune response. The pituitary, with rich blood supply, is vulnerable. Previous studies have observed changes in adrenocorticotropic hormone and cortisol in patients treated with sintilimab, further confirming the diagnosis of immune-related hypophysitis in this case (18, 19) (3). After discontinuing sintilimab and undergoing hormone therapy, the patient’s thirst decreased, vision improved, diabetes insipidus was relieved, and adrenocorticotropic hormone returned to normal (4). The concurrent medications, temozolomide and bevacizumab, have mechanisms that do not seem to trigger immune-related hypophysitis, and no relevant reports were found, excluding their influence (20, 21). In summary, the association rating between sintilimab and immune-related hypophysitis is “likely,” with a score of 6 points according to the Naranjo evaluation principle, indicating a strong association (22) (**Table 2**).

**Table 1:** Evaluation of the Association of Adverse Drug Reactions.

No.	Index	Results
1	Is there a reasonable time relationship between drug use and the occurrence of adverse reactions/events?	+
2	Does the reaction conform to the known types of adverse reactions of this drug?	?
3	After drug withdrawal or dose reduction, does the reaction disappear or lessen?	+
4	Does the same reaction event recur when the suspected drug is used again?	?
5	Can the reaction/event be explained by the effects of concomitant drugs, the progress of the patient’s condition, and the influence of other treatments?	–

+ means yes; - means no; ? means unknown.

**Table 2:** Naranjo Adverse Drug Reaction Assessment Scale.

Related Questions	Question Scores			Answers	Scores
	Yes	No	Unknow		
1. Are there conclusive reports on Sintilimab-related Autoimmune Hypophysitis?	+1	0	0	No	0
2. Did Autoimmune Hypophysitis occur after the use of Sintilimab?	+2	-1	0	Yes	+2
3. Did Autoimmune Hypophysitis resolve after discontinuation of Sintilimab?	+1	0	0	Yes	+1
4. Did Autoimmune Hypophysitis recur after re-use of Sintilimab?	+2	-1	0	Unknow	0
5. Are there other causes that could cause Autoimmune Hypophysitis?	-1	+2	0	No	+2
6. Does this adverse reaction recur after the application of placebo?	-1	+1	0	Unknow	0
7. Does Sintilimab reach a toxic concentration in the blood or other body fluids?	+1	0	0	Unknow	0
8. Does Autoimmune Hypophysitis worsen with an increase in the dose of Sintilimab or relieve with a decrease in the dose?	+1	0	0	Unknow	0
9. Has the patient been exposed to similar drugs and had a similar reaction?	+1	0	0	No	0
10. Is there objective evidence of this adverse reaction with the patient’s Autoimmune Hypophysitis?	+1	0	0	Yes	+1
Total score	+6				

A total score of ≥9 indicates a definite association between the drug and the adverse reaction. A total score between 5 and 8 indicates a high probability of an association between the two. A total score of 1-4 indicates that there may be some association. A total score of ≤0 indicates that this association is suspect.

### 3.4 Treatment measures for sintilimab-associated immune-related hypophysitis

The occurrence of immune-related hypophysitis is associated with excessive immune activation and is an autoimmune reaction. According to literature searches, the main treatment measures include (1): Glucocorticoid therapy: For patients with headache, visual impairment, or pituitary dysfunction, moderate-dose glucocorticoid therapy is typically used, such as prednisone. Initially, a higher dose is usually adopted, which is then gradually tapered based on the patient’s response and discontinued after symptom improvement (2). Hormone replacement therapy: For patients with pituitary insufficiency, corresponding hormone replacement therapy is necessary to maintain normal endocrine function (3). Immunosuppressants: In some refractory or recurrent cases, immunosuppressants such as cyclophosphamide or azathioprine may be required (4). Monitoring and follow-up: During treatment, regular monitoring of the patient’s pituitary hormone levels is necessary, and changes in pituitary morphology should be assessed through magnetic resonance imaging (MRI) to guide treatment decisions.

In summary, as an effective immunotherapy drug for cancer, sintilimab-induced immune-related hypophysitis, although rare, requires close attention from clinicians due to its insidious nature and association with immunotherapy. For non-small cell carcinoma patients with pituitary metastasis, special attention should be paid to the patient’s urine volume and visual changes during diagnosis and treatment to detect hypophysitis promptly and intervene early. Additionally, patients should have regular follow-ups after treatment to monitor symptom changes and pituitary imaging, and when necessary, undergo MRI and hematological examinations to ensure timely identification and management of potential complications, thereby improving treatment outcomes and quality of life.

## 4 Conclusion

When using sintilimab to treat non-small cell lung cancer, close attention should be paid to patient symptoms, and early intervention with glucocorticoids should be considered if immune-related hypophysitis occurs.

## Data Availability

The original contributions presented in the study are included in the article/supplementary material. Further inquiries can be directed to the corresponding author.
